# The role of CD8 + T lymphocytes in chronic obstructive pulmonary disease: a systematic review

**DOI:** 10.1007/s00011-020-01408-z

**Published:** 2020-10-10

**Authors:** Maya Williams, Ian Todd, Lucy C. Fairclough

**Affiliations:** School of Life Sciences, The University of Nottingham, Life Sciences Building, University Park, Nottingham, NG7 2RD UK

**Keywords:** Chronic obstructive pulmonary disease, CD8 + T lymphocyte, Smoking, Cytotoxic T cell, Lung disease

## Abstract

**Objective and design:**

This systematic review aims to establish the role of CD8 + T lymphocytes in COPD.

**Methods:**

Forty-eight papers published in the last 15 years were identified for inclusion.

**Results:**

CD8 + T-cells are increased in the lungs of patients with COPD (17 studies, 16 positive) whereas in the circulation, findings were inconclusive. Activation of CD8 + T-cells was enhanced in lungs (four studies, three positive) but cell phenotype was unclear. There was substantial evidence of a higher proportion of type 1 CD8 + (Tc1) cells in COPD (11 studies, 9 positive), though the population of type 2 (Tc2) cells was also increased (5 studies, 4 positive). CD8 + T-cells in COPD exhibited greater expression of cytotoxic proteins (five studies, five positive). Studies assessed a variety of questions so evidence was insufficient to draw firm conclusions. The role of CD8 + T-cells at acute exacerbation of COPD and also their contribution to alveolar destruction can only be hypothesised at this stage.

**Conclusions:**

Not only is the number of CD8 + T-cells increased in COPD, these cells have increased capacity to exert effector functions and are likely to contribute to disease pathogenesis. Several mechanisms highlighted show promise for future investigation to consolidate current knowledge.

**Electronic supplementary material:**

The online version of this article (10.1007/s00011-020-01408-z) contains supplementary material, which is available to authorized users.

## Introduction

COPD (chronic obstructive pulmonary disease) is a treatable and preventable disease state, characterized by progressive airflow limitation that is not fully reversible. It is a current and growing cause of mortality and morbidity worldwide. The pathological hallmarks of COPD are destruction of the lung parenchyma (pulmonary emphysema), inflammation of the central airways (chronic bronchitis) and inflammation of the peripheral airways (respiratory bronchiolitis). The destructive changes and tissue remodelling observed in COPD are a result of complex interactions between cells of the innate and adaptive immune systems, with growing interest in the role of CD8 + T lymphocytes.

By collating the current evidence, this systematic review aims to determine the exact role that CD8 + T lymphocytes have in the pathogenesis of COPD. Since the burden of COPD is increasing, a better understanding of the underlying pathology could help to identify new therapeutic targets.

## Methods

### Search strategy

All searches were carried out in October 2019. PubMed and Embase were the two databases chosen for this systematic review. Controlled vocabulary was included following a search of the MeSH and Emtree databases, respectively.

The search strategy for PubMed was as follows:

(((T-lymphocytes, Cytotoxic [MeSH Terms]) OR CD8-positive T-lymphocytes [MeSH Terms])) AND (((((pulmonary disease, chronic obstructive [MeSH Terms]) OR pulmonary emphysema [MeSH Terms]) OR chronic bronchitis [MeSH Terms]) OR COPD [Title/Abstract]) OR chronic obstructive pulmonary disease [Title/Abstract]) 181 papers were identified as a result of this search.

The search strategy for Embase was as follows:

(exp chronic obstructive lung disease/OR exp lung emphysema/OR exp chronic bronchitis/OR chronic obstructive pulmonary disease.ab. OR chronic obstructive pulmonary disease.ti. OR COPD.ab. OR COPD.ti.) AND (exp cytotoxic T lymphocyte/ OR exp CD8 + T lymphocyte/).

617 papers were identified as a result of this search.

### Inclusion of publications

The database searches revealed a total of 798 papers which was reduced further to 710 following removal of duplicates. Title and abstract screening of the remaining papers was conducted using Rayyan systematic review scanner software. Common reasons for exclusion included publications which did not consider COPD or CD8 + T lymphocytes specifically and those of the wrong publication type, such as review articles. Further to this, papers written in a foreign language were excluded due to the potential loss of meaning in translation. The decision to place a time restriction to the last 15 years (2005 onwards) was made to only include the most topical research. Consequently, 51 papers were identified for full text analysis.

**Fig. 1:**
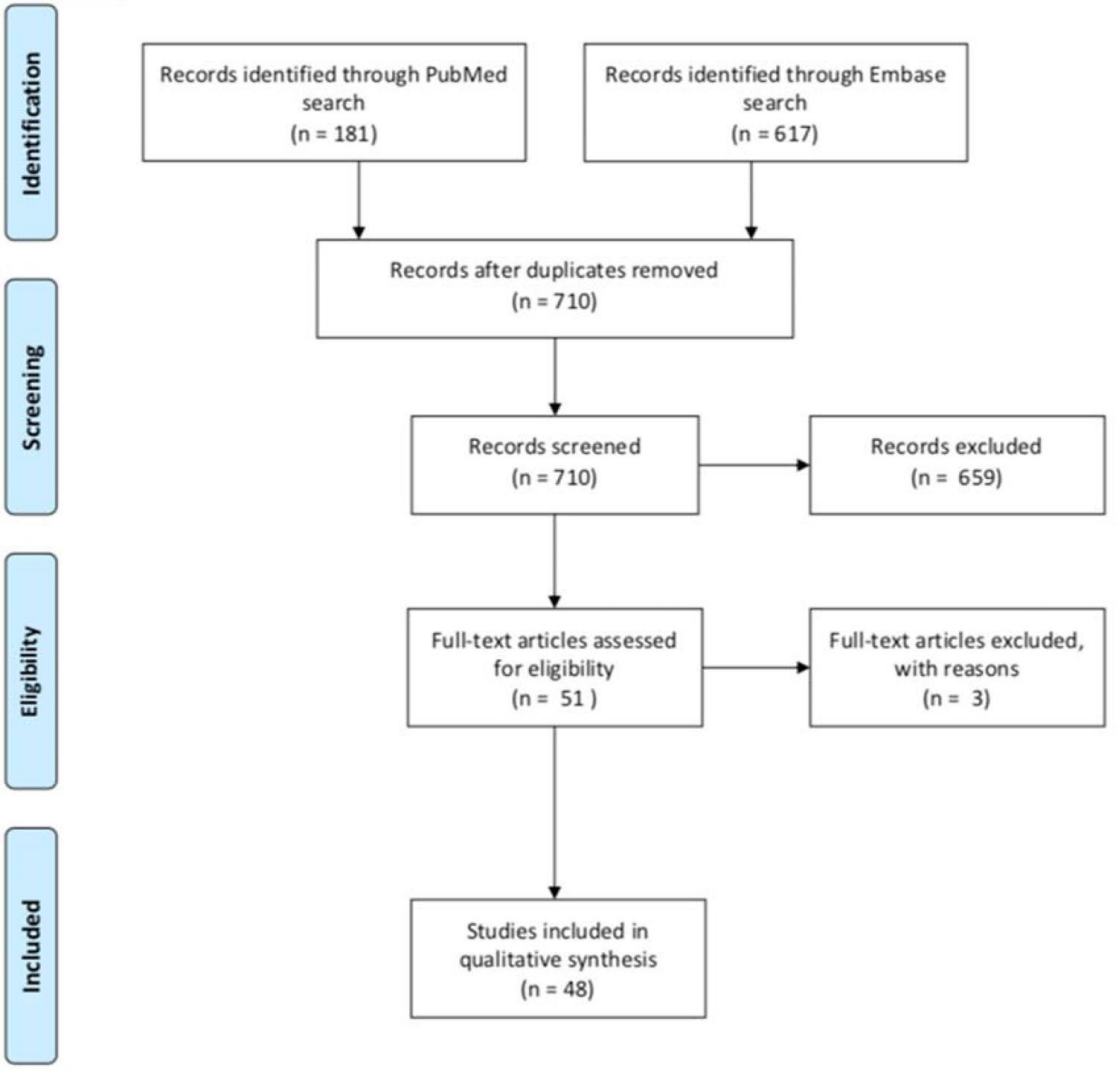
PRISMA 2009 flow diagram showing the screening process and the inclusion of studies

Three publications were excluded during full text analysis, of which two were conference abstracts whereas one was not in English. A final total of 48 papers were included in the study. The PRISMA 2009 flow chart of article selection is shown in Fig. 1.

Further details of the search strategy and inclusion/exclusion criteria for this systematic review are given in Online Resource 1. Following full text screening, 48 papers were identified for inclusion.

## Results

### Presence of CD8 + T lymphocytes in COPD

Ten studies analysed the presence of CD8 + T lymphocytes in patients with COPD [1–10], nine of which noted a significant association (further details are given in Online Resource 2). Eight studies examined cells in lung samples, with four of these also examining cell numbers in peripheral blood, and two studies examined cells in peripheral blood alone. All included human patients with COPD and both non-COPD smokers (S) and healthy non-smokers (HNS) were included in eight of the studies, whilst one had no control and another included asthmatics and non-smokers as comparators.

Of the four studies which sampled lung tissue [1–3, 8], all used immunohistochemistry to show increased quantities of CD8 + T lymphocytes in COPD. Analysis of BAL was carried out in three studies [7, 9, 10], one of which also sampled induced sputum [9]. Like the above studies, evidence for increased numbers of CD8 + T lymphocytes in COPD was presented in all three. Samples obtained by endobronchial biopsy were included in two studies [2, 4]. Löfdahl et al. [4] showed a significantly greater number of intraepithelial CD8 + T lymphocytes in COPD patients compared to HNS. On the other hand, Eapen et al. [2] observed lower numbers of CD8 + T lymphocytes in the large airways of smokers with and without COPD.

Hodge et al. [10] performed bronchial brushing and reported that the percentage of bronchial mucosal CD8 + T lymphocytes was significantly increased in smoker controls and also in the group of ex-smokers with COPD taking inhaled corticosteroids.

Of the six studies which analysed peripheral blood [5–10], three reported no difference in the CD8 + T lymphocyte counts between subjects with COPD, S or HNS [6, 8, 9]. On the contrary, Forsslund et al. [7] reported a lower percentage of CD8 + T lymphocytes in the peripheral blood of smokers with and without COPD compared to HNS. This is contradictory to the findings of Hodge et al. [10] who showed an increased percentage of CD8 + T lymphocytes in the peripheral blood of both ex-smoking and current smoking patients with COPD in comparison to S and HNS.

Fifteen further studies included findings regarding the numbers of CD8 + T lymphocytes in COPD. However, the main findings from all 15 studies are described elsewhere in this review [11, 15–17, 19, 21, 25, 28–30, 33, 36–39].

### Phenotypic analysis of CD8 + T lymphocytes in COPD

Investigation into surface marker expression/phenotype of CD8 + T lymphocytes in COPD was conducted in 11 studies [11–21] (further details are given in Online Resource 3).

CD69, CD25 and HLA-DR are expressed on activated CD8 + T lymphocytes. Three studies reported increased activation of CD8 + T lymphocytes in COPD lung samples [11, 12, 16] and two highlighted a smoking-independent activation of CD8 + T lymphocytes in COPD [11, 12]. In contrast to the above, a small study [14] reported negligible and indifferent CD25 expression on BAL CD8 + T lymphocytes in both COPD and controls. Two studies highlighted changes in KIR expression of CD8 + T lymphocytes in COPD patients and S [12, 16].

Two studies [14, 18] investigated CD103, an integrin thought to have co-stimulatory effects on T cell activation and a role in adhesion. Neither reported a difference in CD103 expression on circulating CD8 + T lymphocytes between COPD and controls. Glader et al. [14] also found no difference in the lungs whereas Mikko et al. [18] noted an increase of CD8 + /CD103 + T cells in BAL of current smokers with COPD and S compared to HNS.

Seven studies characterised the phenotype of CD8 + T lymphocytes in COPD. In the lung, two of five studies showed that a higher proportion of CD8 + T lymphocytes are of the memory phenotype (CD45RO +) in COPD [14, 19]. Urbanowicz et al. [19] also highlighted an elevated percentage of cytotoxic effector memory (TEMRA) cells in induced sputum of COPD patients. Similarly, Barceló et al. [13] concluded a final-activation maturation state of CD8 + CD45RA + T lymphocytes in the lung following analysis of BAL. Mikko et al. [18] also reported that intraepithelial CD8 + T lymphocytes were terminally differentiated. Conversely, Freeman et al. [20] concluded that CD8 + T lymphocytes in lung tissue of COPD patients were short-term effector memory T cells (CD62L-/CD27-) rather than terminally differentiated. Meanwhile in peripheral blood, one study [13] found no difference in the proportions of naïve and memory CD8 + T lymphocytes between COPD and control groups. Koch et al. [17] observed a smoking-related cytotoxic effector phenotype (CD27-/CD45RA +) on CD8 + T lymphocytes in smokers with and without COPD compared to HNS whereas Urbanowicz et al. [21] on the other hand detected a *lower* proportion of TEMRA cells in COPD and S. Finally, three studies demonstrated increased expression of chemokine receptors on CD8 + T lymphocytes in COPD [15, 17, 19].

Two studies found that upregulation of the CXCR3 chemokine receptor on CD8 + T lymphocytes in COPD was smoking-independent [17, 19]. This increase was observed in both peripheral blood and induced sputum. On the contrary, analysis of BAL by Smyth et al. [15] found no difference in CXCR3 but did find increased expression of CCR5 and CCR3 on CD8 + T lymphocytes from the lungs of COPD compared to controls.

### Inflammatory cytokine profile of CD8 + T lymphocytes in COPD

Eleven studies investigated the inflammatory cytokine profile of CD8 + T lymphocytes in COPD [22–32]. One study used a mouse model [31] whilst the remaining obtained human samples (further details are given in Online Resource 4).

Greater production of IFN-g and/or TNF-a by CD8 + T lymphocytes from peripheral blood COPD samples was shown in five studies [10, 23–26]. As for the lung samples, two studies showed enhanced production of Tc1 cytokines by CD8 + T lymphocytes obtained from COPD patients [10, 32], being higher in smokers than ex-smoker patients. Moreover, two studies highlighted the effects of cigarette smoke in induction of a Tc1 phenotype [27, 31]. By contrast, two studies found no association between the Tc1 profile of CD8 + T lymphocytes and COPD [29, 30].

Five studies investigated the Tc2 subpopulation of CD8 + T lymphocytes in COPD [23, 25, 26, 29, 30]. Two of these studies examined BAL and highlighted an increase in Tc2 cytokine expression by CD8 + T lymphocytes in COPD [29, 30]. In peripheral blood, two studies highlighted a marked increase in Tc2 cells amongst CD8 + T lymphocytes in COPD [25, 26]. Another study also showed a trend towards increased proportions of IL-4 producing CD8 + T lymphocytes in COPD, but this was not significant [23]. On the other hand, Barceló et al. [29] found no difference in cytokine expression by circulating CD8 + T lymphocytes in COPD or controls whereas Barcyzk et al. [30] reported that the number of IL-4 producing CD8 + T lymphocytes was lower in the peripheral blood than in BAL.

Two studies calculated the Tc1/Tc2 ratio in participant samples: Shirai et al. [26] reported no difference in the ratio of IFN-g producing/IL-4-producing CD8 + T lymphocytes between COPD subjects and controls following analysis of peripheral blood. Conversely, Yu et al. [28] reported a significantly higher Tc1/Tc2 ratio in both BAL and peripheral blood COPD samples. They also showed that a dominant Tc1 phenotype is associated with decreased lung function.

Finally, of the three studies which investigated the Tc17 phenotype of CD8 + T lymphocytes in COPD, two studies utilised peripheral blood [23, 24] whereas one study sampled bronchial mucosa [22]. One of the peripheral blood studies [23] found no difference in the proportion of IL-17A and IL-17F producing CD8 + T lymphocytes in the circulation of COPD and control samples. On the contrary in the lung, Chang et al. [22] confirmed by immunofluorescence and reverse transcription PCR that bronchial CD8 + T lymphocytes from COPD subjects expressed IL-17A and IL-17F. Further investigation showed that CD8 + T lymphocytes were the principal IL-17A and IL-17F producing cells. Similarly, analysis of peripheral blood by Xu et al. [24] showed that a significantly higher percentage of CD8 + T lymphocytes were of the Tc17 phenotype in COPD.

### Cytotoxic function of CD8 + T lymphocytes in COPD

Ten studies investigated the cytotoxic function of CD8 + T lymphocytes in COPD [19–21, 33–39]. All included human participants and one study also used a mouse model [39] (further details are given in Online Resource 5). Analysis of human lung tissue was undertaken in four studies [20, 33, 34, 38], peripheral blood in a different four studies [21, 36, 37, 39] and samples of induced sputum [19] and epithelial lining fluid [35] were obtained in one study each.

Seven studies investigated the expression cytotoxic mediators by CD8 + T lymphocytes, of which six showed increased expression of granzyme and/or perforin in COPD [19, 20, 33–35, 39] whereas one reported decreased expression [21]. All three studies which analysed human lung tissue found a significant association [20, 33, 34]. Analysis of induced sputum by Urbanowicz et al. [19] (see Table 2) showed increased expression of both perforin and granzyme B on CD8 + T lymphocytes from COPD. Furthermore, Shiratuschi et al. [35] reported increased perforin in the central and peripheral airways of COPD following analysis of epithelial lining fluid.

Of the two studies which sampled peripheral blood, one observed significantly *lower* expression of perforin and granzyme B by CD8 + T lymphocytes in COPD compared to controls [21] (see Table 2). Hodge et al. [39] on the other hand showed increased numbers of CD8/CD28^null^ cells in COPD and that these cells express significantly more granzyme B and perforin than CD8/CD28 + T cells. Using a mouse model they showed that cigarette smoke exposure induced a significant increase in cytotoxic CD8/CD28^null^ T lymphocytes in BAL and a trend towards an increase in lung tissue and peripheral blood.

Three studies evaluated the Fas/FasL pathway of cytotoxicity on CD8 + T lymphocytes. Two studies [36, 37] examined peripheral blood and showed increased expression of Fas (CD95) on circulating CD8 + T lymphocytes from COPD samples compared to smoker and non- smoker controls. One study [36] also showed that the proportion of CD8 + /Fas + T lymphocytes was associated with both reduced lung function and hypoxaemia. On the contrary in the lung, Freeman et al. [20] (see Table 2) found no association between lung function and the expression of FasL mRNA transcripts by lung CD8 + T lymphocytes.

Expression of PD-1, a member of the CD28 family of TCR molecules which is associated with a loss of cytotoxic function, was shown in one study to be increased on CD8 + T lymphocytes from COPD lung samples [38].

### Other effector functions of CD8 + T lymphocytes in COPD

Five studies investigated other effector functions of CD8 + T lymphocytes in COPD. Four were mouse models [40–43] and one human [44]. Lung tissue was sampled in all four mouse models, three of which also examined BAL [41–43], whereas lung tissue, BAL and peripheral blood were sampled in the human study (further details are given in Online Resource 6).

Dysregulation of the CD8 + T cell receptor (TCR) was found in two studies [40, 44] and the indirect effects of CD8 + T lymphocytes in the inflammatory process of COPD were shown in three murine models [41–43].

### Role of CD8 + T lymphocytes in acute exacerbations of COPD

There were four papers identified which highlighted the role of CD8 + T lymphocytes in acute exacerbations of COPD (AECOPD) [45–48] (further details are given in Online Resource 7). Two studies which analysed peripheral blood had contrasting conclusions. When comparing the same subjects at stable condition and at AECOPD, Freeman et al. [46] showed that CD8 + T lymphocytes decreased significantly in AECOPD and then increased back to a constant level at stable condition. They also concluded that the decline in CD8 + T cell frequency could be a potential marker of AECOPD since it preceded the onset of symptoms. Conversely, Chen et al. [45] compared three subject groups and highlighted that the percentage of CD8 + T lymphocytes in the peripheral blood was significantly greater in AECOPD than in stable COPD and non-smoking control samples.

There was overall agreement that the percentage of CD8 + T lymphocytes increased in the lungs of patients at onset of AECOPD. This was reported by two studies, both of which took induced sputum samples at the initial onset of exacerbation and then again after a follow-up of 8 or 16 weeks [47, 48].

## Discussion

Evidence from this review indicates that CD8 + T lymphocytes are both increased in number and have increased functional activity in COPD and highlights some potential mechanisms by which they may elicit pathogenesis.

The evidence shows that that patients with COPD have increased numbers of CD8 + T lymphocytes in the lung. This was highlighted in 16 out of 17 studies following analysis of lung tissue, BAL, induced sputum or bronchial mucosa. The same conclusion was not reached for the circulation where 7 of the 14 studies found no difference, 5 showed an increase and 2 found a decrease in the number of cells in the peripheral blood of patients with COPD compared to controls. Systemic inflammation is present to some extent in COPD so it is interesting that the findings are so inconclusive. Perhaps the CD8 + T lymphocytes migrate from the blood to the lungs during COPD or perhaps they spill out from the lungs into the blood [49] or combination of the two. Most likely however is that the inconsistency could be attributed to variability between studies: for instance, the control participants were different in different studies, meaning that some COPD groups were compared to smokers whilst others were compared to healthy non-smokers.

Some studies showed a smoking related increase in CD8 + T lymphocytes however others did not. This suggests that the increased presence of these cells is most closely related to disease, not simply in response to cigarette smoke. The evidence also suggests that the number of cells in the lung is associated with disease severity. However, not all studies investigated the association between cell frequency and cigarette smoking and/or disease severity and therefore evidence is insufficient to reach a firm conclusion.

In addition to a greater quantity of cells, there was evidence showing increased activation of CD8 + T lymphocytes in the lungs in COPD. The only study [14] which found no difference in activation had the smallest sample size in the review with only ten subjects, therefore making it less generalisable to the COPD population. In two studies, increased activation of lung CD8 + T lymphocytes was shown to be smoking-independent, raising the question as to what stimulus these cells are responding to. Activation occurs following recognition and binding to antigen peptides but is also mediated by cytokines such as IL-12 and IL-18 [50].

Upregulation of chemokine receptors on CD8 + T lymphocytes was also shown in COPD. CXCR3 is highly expressed on effector T cells following activation by ligands such as IP-10 [51]. CD8 + T lymphocytes themselves promote production of IP-10 via IFN-g [52]. IP-10 then recruits more CXCR3 + CD8 + T lymphocytes to the lung where they exert their inflammatory and destructive effects whilst continuing to attract more cells.

Effector CD8 + T lymphocytes differentiate into subpopulations which can be discriminated by cytokine profile. Nine of eleven studies found a higher proportion of pro-inflammatory Tc1 cells in COPD. The two which did not reach this consensus were the oldest and therefore consistency in the recent findings increases confidence in this conclusion.

Likewise, conclusive findings from three mouse models showed significantly reduced alveolar destruction in the absence of CD8 + T lymphocytes, further suggesting their effects involve mediating emphysema-like changes. Animal models however are not truly representative of human disease and so the extent and means in which CD8 + T lymphocytes induce alveolar destruction in human tissue requires further investigation.

It was found consistently in five studies that in patients with COPD, CD8 + T lymphocytes have elevated expression of granzymes and/or perforin in the lung, following analysis of lung tissue, epithelial lining fluid or induced sputum. Expression of granzyme B has been correlated with apoptosis of bronchial epithelial cells [53]. Therefore, it is plausible that increased expression of cytotoxic mediators by the already increased number of CD8 + T lymphocytes contributes to lysis of structural cells in the lung, which is a principal characteristic of emphysema. Nevertheless, CD8 + T lymphocytes may not be the only source of cytotoxic proteins. Two of the three studies which sampled lung tissue also noted staining of granzyme B on resident lung cells and innate immune cells, therefore suggesting these cells are not solely responsible for direct cell lysis.

In addition, there was evidence of increased Tc2 cells in COPD in 2/2 studies which examined BAL and 2/5 studies of the peripheral blood. Tc2 cells express IL-4 and IL-13 which promote immunoglobulin production by B lymphocytes [54]. Therefore, a possible consequence of increased Tc2 cells is greater antibody production. Tc2 cells have been associated with autoimmune diseases such as rheumatoid arthritis [55] so it is not out of the question that antibody production against self-antigens might contribute to the persistent inflammatory response in COPD. There is also literature describing increased autoantibodies in COPD [56], but the autoimmune function of CD8 + T lymphocytes specifically is yet to be explored.

AECOPD is a common cause of hospitalisation so a better understanding of the immune response could provide insight for diagnostic and pharmacological targets. The role of CD8 + T lymphocytes in AECOPD was insufficiently explored but showed some promise. The number of CD8 + T lymphocytes in the lung, the majority of which were Tc2, was increased at AECOPD. This is unsurprising since AECOPD are associated with infection and so an enhanced immune response would be expected [57]. One cohort study which analysed peripheral blood found a decrease in CD8 + T lymphocytes in the circulation at AECOPD which then increased to a constant level at stable condition [46]. This may reflect cell extravasation out of the blood to fight infection in the lung. Interestingly, the decline in CD8 + T lymphocytes preceded the onset of symptoms, therefore highlighting these cells as a potential biomarker for AECOPD. Further investigations would be required to confirm this, but since diagnosis is based mainly on symptoms, there is demand for a clinical biomarker, making this a potentially exciting concept.

There are a number of unavoidable limitations associated with this systematic review: these are detailed in the Supplementary Information. Briefly, differences between studies in terms of design (e.g., cross-sectional versus longitudinal), patient definition (e.g., current and ex-smokers), human versus animal studies, and heterogeneity in outcomes measured must be acknowledged.

## Conclusion

This review confirms that the number of CD8 + T lymphocytes is increased in the lungs of patients with COPD. It also highlights the increased capacity of these cells to exert effector functions; namely secretion of pro-inflammatory cytokines and expression of cytotoxic proteins, and goes on to outline several potential mechanisms for their role in disease pathogenesis. The lack of evidence in some areas made it impossible to draw firm conclusions at this stage, but also identified some promising areas for further investigation.

## Electronic supplementary material

Below is the link to the electronic supplementary material.

Supplementary file1 (PDF 56 kb)

Supplementary file2 (PDF 82 kb)

Supplementary file3 (PDF 86 kb)

Supplementary file4 (PDF 86 kb)

Supplementary file5 (PDF 72 kb)

Supplementary file6 (PDF 77 kb)

Supplementary file7 (PDF 67 kb)
