# Supplementary material for: The role of CD8 + T lymphocytes in chronic obstructive pulmonary disease: a systematic review
Source: Inflamm Res. 2020 Oct 10;70(1):11–8. doi: 10.1007/s00011-020-01408-z (PMC7806561; doi:10.1007/s00011-020-01408-z)
Supplement: Supplementary file 1 — Supplementary file1 (PDF 56 kb) [file 11_2020_1408_MOESM1_ESM.pdf]

ONLINE RESEOURCE 1

ELECTRONIC SUPPLEMENTARY MATERIAL (ESM-1)

INFLAMMATION RESEARCH

The role of CD8+ T lymphocytes in chronic obstructive pulmonary disease: a systematic review.

Maya Williams, Ian Todd, Lucy C. Fairclough

**Corresponding author: Dr Lucy C. Fairclough, School of Life Sciences, The University of Nottingham, Life Sciences Building, University Park, Nottingham NG7 2RD, United Kingdom.
Email: lucy.fairclough@nottingham.ac.uk**

METHODS

Prior to starting this systematic review, an initial search of the Cochrane database showed no indication that a review of this topic had been undertaken previously.

Eligibility criteria for consideration of studies:

Publications considered in this review intended to answer the question:
"What is the role of CD8+ T lymphocytes in the pathogenesis of chronic obstructive pulmonary disease?"

Types of studies:

All of the publications considered were laboratory based and a large proportion followed a cohort or cross sectional study design involving both COPD patients and control groups.

Types of participants:

Most studies included human subjects and of these, all specified the inclusion patients with COPD. Diagnosis of COPD was most frequently determined using the GOLD criteria or American Thoracic Society (ATS)/European Respiratory Society (ERS) guidelines but was not specified in all papers. Studies focusing on patients with COPD, chronic bronchitis and emphysema were included but other respiratory diseases were not.

Asymptomatic smokers and healthy non-smokers were most commonly chosen as comparators to patients with COPD, though the exact criteria for such controls varied between studies. Furthermore, the long-term nature of the disease meant that the use of treatment was not a reason for exclusion. The decision to include relevant animal studies was made since their findings offer perspective from a controlled laboratory environment.

To gain a comprehensive understanding of the overall effects of CD8+ T lymphocytes in COPD, samples from both the lungs, such as tissue explants, bronchoalveolar lavage fluid (BAL) and induced sputum, and from peripheral blood were included.

Types of outcome measures:

Explicit analysis of CD8+ T lymphocytes was required for inclusion and consequently the presence of such cells in lung and systemic compartments was frequently compared between COPD patients and controls. Further investigation determined associations between the clinical presentation of disease, commonly defined by GOLD stage, and the frequency, function and/or phenotype of CD8+ T lymphocytes.

Other information sources

Grey literature was excluded during the screening process since it encompasses material which has been "published outside of traditional commercial publishing" as defined by the Cochrane guidelines. Examples include conference proceedings, dissertations and literature which has not yet been peer-reviewed. Nevertheless, exclusion of grey literature increased susceptibility to publication bias in favour of positive results since negative findings are less likely to be published.

Limitations associated with this systematic review

Since many included studies were cross-sectional, the outcome may not be truly representative and it is also impossible to determine a temporal relationship between lung function and the outcome studied. Furthermore, comparison of studies deemed to have the 'same' outcome measure was limited by heterogeneity in study design and conduct. For instance, cohort studies included in this review examined the same group of patients longitudinally whereas cross- sectional studies took a snapshot of the populations studied at any one time, therefore making it difficult to compare, even if the outcome studied was the same.

Across studies, variability in the COPD group included but was not limited to the sample population size, diagnostic criteria and smoking status. Some studies defined patients as current or ex-smokers, though the criteria for 'ex-smokers' also varied, whilst others did not give details. Likewise the criteria for control populations, usually defined as smokers or non- smokers without decreased lung function, were inconsistent throughout. It is reasonable to assume that reporting a significant difference for the COPD group would be affected by whether the control group was made up of smokers or non-smokers since smoking is such an established risk factor, but the variation amongst studies often made it impossible to establish the true effect of smoking.

Also, the inclusion of animal models was useful for investigating disease mechanisms in a controlled environment but assumptions cannot be made for human disease.

Moreover, there was diversity in the outcome being measured in the identified studies. However, since the review investigated the role of CD8+ T lymphocytes in COPD, it was inevitable that included studies would investigate a variety of questions and therefore have different outcome measures. Consequently there was a paucity of studies that evaluated some aspects; CD8+ T lymphocytes at acute exacerbation and also analysis of certain cell subsets or specific cell surface molecules just to name a few. It is therefore only possible to hypothesise in these instances.

Limitations in the methodology of this study must also be acknowledged. The decision not to include grey literature increased the risk of publication bias since findings from such sources may not be invalid simply because they have not been published. Moreover, any papers not written in English were excluded, thereby increasing susceptibility to language bias.
