# Supplementary material for: The role of CD8 + T lymphocytes in chronic obstructive pulmonary disease: a systematic review
Source: Inflamm Res. 2020 Oct 10;70(1):11–8. doi: 10.1007/s00011-020-01408-z (PMC7806561; doi:10.1007/s00011-020-01408-z)
Supplement: Supplementary file 2 — Supplementary file2 (PDF 82 kb) [file 11_2020_1408_MOESM2_ESM.pdf]

ONLINE RESEOURCE 2

ELECTRONIC SUPPLEMENTARY MATERIAL (ESM-2)

INFLAMMATION RESEARCH

The role of CD8+ T lymphocytes in chronic obstructive pulmonary disease: a systematic review.

Maya Williams, Ian Todd, Lucy C. Fairclough

Corresponding author: Dr Lucy C. Fairclough, School of Life Sciences, The University of Nottingham, Life Sciences Building, University Park, Nottingham NG7 2RD, United Kingdom.
Email: lucy.fairclough@nottingham.ac.uk

Table S1: Studies investigating the presence of CD8+ T lymphocytes in COPD. Ten human studies investigated the number of CD8+ T lymphocytes in COPD.

Publication	Title	Subjects	COPD diagnosis	Sample type	Conclusions
Baraldo et al [1]<br><br>Human 2015	Immune activation in a1 anti-trypsin deficiency emphysema (AATD). Beyond the protease-antiprotease paradigm	10 AATD,<br>26 COPD, 17 control smokers (S), 25 healthy non-smokers (HNS)	AATD – serum levels and genotyping COPD – macroscopic quantification of emphysema, method of Heard	Explanted lung samples	Those with usual COPD had a significantly greater number of CD8+ T cells than S and HNS<br>Those with AATD had significantly greater number of CD8+ T cells than HNS<br><br>Suggests AATD is similar to usual COPD
Eapen et al [2]<br><br>Human 2017	Profiling cellular and inflammatory changes in the airway wall of mild to moderate COPD	Large airway: 13 COPD- current smokers (CS), 14 COPD-ex smokers (exS), 20 S, 25 HNS<br><br>Small airway: 9 COPD-CS, 10 COPD exS, 11 S, 10 HNS	COPD – Global Initiative for Obstructive Lung Diseases (GOLD) criteria<br><br>COPD – GOLD stage I/II	Endobronchial biopsy<br><br>Lung tissue	Large airway: CD8+ T cells were the dominant lymphocyte<br>CD8+ T cells were lower than normal in smoker/COPD groups but there was a change in the CD8+/CD4+ T cell ratio.<br>In COPD-CS there was a marked relative excess of CD8+ over CD4+<br><br>Small airway: CD8+ T cells were higher than normal across all clinical groups and especially COPD-CS
Siena et al [3]<br><br>Human 2011	Reduced apoptosis of CD8+ T-lymphocytes in the airways of smokers with mild/ moderate COPD	All COPD smokers: 5 mild, 11 moderate, 3 severe, 6 very severe COPD 16 S, 16 HNS	COPD – GOLD classification I-IV	Lung tissue specimens	The median percentage of apoptotic CD8+ T cells in the submucosa of both the central and peripheral airways was significantly lower in mild/moderate COPD than in HNS, S and severe/very severe COPD When all subjects are considered, no correlation exists between the apoptosis of CD8+ T cells and functional parameters but when severe/very severe COPD are excluded, there is a significant positive correlation between the percentage of apoptotic CD8+ T cells and FEV_1 and FEV_1/FVC in both the central and peripheral airways A significant negative correlation was determined between total CD8+ T lymphocytes and the percentage of apoptotic CD8+ T cells in central and peripheral airways

Lofdahl et al [4]<br><br>Human 2008	Increased intraepithelial T-cells in stable COPD	22 COPD, 14 S, 15 HNS	COPD – GOLD	Endobronchial mucosal biopsy sample	The number of intraepithelial CD8+ T cells per millimetre of epithelium was significantly higher in COPD compared to HNS, but no significant difference was determined when compared to S
Chen et al [5]<br><br>Human 2012	Imbalance of circulating T-lymphocyte subpopulation in COPD and its relationship with CAT performance	All COPD: 13 moderate, 14 severe, 25 very severe	American Thoracic Society/ European Respiratory Society (ATS/ERS) standards<br><br>COPD Assessment Test (CAT score)	Peripheral blood	Proportion of CD8+ T cells in the group with CAT>30 was significantly higher than other groups. There was no significant difference in the proportion of CD8+ T cells in groups with 20<score£30, 10<score£20 and score£10<br><br>Positive correlation between the proportion of CD8+ T cells and CAT score when score>20 but not when score£20<br><br>No difference in the proportion of CD8+ T cells in the peripheral blood between moderate, severe, extremely severe COPD when comparing by GOLD stage
Mathai et al [6]<br><br>Human 2013	Peripheral blood T-cell populations in COPD, asymptomatic smokers and healthy non-smokers in Indian subpopulation – a pilot study	21 COPD, 19 S, 20 HNS	History of cough with sputum and/or dyspnoea on most days of the month for at least 3 months for at least 2 consecutive years, and airflow limitation $FEV_1/FVC < 0.70$ and $FEV_1 < 80\%$ predicted	Peripheral blood	There was no difference in the absolute counts of peripheral blood T cell subsets among HNS, S and COPD<br>There was no difference between the mean CD8+ T cell counts when comparing COPD, S and HNS<br><br>No correlation was determined between CD8+ T cells and smoking intensity, or with the type of smoking (bidi or cigarette)

Forsslund et al [7]<br><br>Human 2014	Distribution of T-cell subsets in BAL fluid of patients with mild to moderate COPD depends on current smoking status and not airway obstruction	27 COPD-CS, 11 COPD-exS, 40 S, 40 HNS	GOLD stage I-II	Bronchoalveolar lavage fluid (BAL)<br><br>Peripheral blood	The percentage of CD8+ T cells was significantly higher in BAL from COPD- CS and S<br>There was a lower percentage of CD8+ T cells in BAL from COPD-exS compared to both COPD-CS and S.<br><br>The percentage of CD8+ T cells in BAL from COPD-CS was positively correlated with the number of cigarettes smoked per day in the last 6 months. This correlation was even stronger in male COPD-CS<br><br>In the peripheral blood, COPD-CS and S groups had a lower median percentage of CD8+ T cells than HNS.
Olloquequi et al [8]<br><br>Human 2010	Differential lymphocyte infiltration in small airways and lung parenchyma in COPD patients	16 moderate COPD, 16 very severe COPD, 18 S, 9 HNS	GOLD stage II (moderate) or GOLD stage IV (very severe)	Excised lungs or lobes<br><br>Peripheral blood	Small airway: COPD had increased density of CD8+ T cells than controls. Subjects with moderate COPD had a significantly higher density of CD8+ T cells in the epithelial layer, and also in the total small airway than HNS. there were no differences in CD8+ T cell density in smooth muscle.<br><br>Lung parenchyma: the CD8+ T cell density was significantly higher in the interstitium of COPD compared to controls. There was no significant difference in CD8+ T cell density in areas proximal and distal to emphysema in COPD lungs.<br><br>Moderate COPD and S groups had significantly higher CD8+ T cells in the bronchial wall compared to parenchymal interstitium<br><br>The number of pack years smoked was positively correlated with CD8+ T cell density in the total small airway, bronchiolar connective compartment and epithelium, and the number of CD8+ T cells in the alveolar interstitium<br><br>Peripheral blood – no difference in cell numbers among 4 clinical groups

Urboniene et al [9]<br><br>Human 2013	Distribution of gd and other T-lymphocyte subsets in patients with chronic obstructive pulmonary disease and asthma	20 COPD, 18 asthma, 14 healthy subjects	GOLD stage II and III	Induced sputum<br>BAL<br>Peripheral blood	The percentage and absolute numbers of CD8+ T cells in induced sputum was significantly higher in COPD compared to asthmatics and healthy subjects.<br>CD8+ T cells were also higher in BAL in COPD compared to asthmatics and healthy subjects. CD8+ T cell number in both BAL and induced sputum correlated negatively with FEV_1<br><br>There was no difference in the number of CD8+ T cells in peripheral blood between any study groups.
Hodge et al [10]<br><br>Human 2007	Increased intracellular T helper 1 pro-inflammatory cytokine production in peripheral blood, bronchoalveolar lavage and intraepithelial T cells of COPD subjects	7 COPD-CS,<br>5 COPD-CS- using inhaled corticosteroids (ics) ,<br><br>7 COPD-exS, 6 COPD-exS- ics, 7 S, 8 HNS	GOLD	BAL<br>Bronchial mucosa<br>Peripheral blood	BAL: percentage of CD8+ T cells was significantly increased in COPD-CCS and S compared to HNS. The percentage of CD8+ T cells producing IFN-g and TNF-a was increased in COPD and S compared to HNS. COPD-CS also had a significantly higher percentage of CD8+ T cells producing IFN-g and TNF-a than COPD-exS<br><br>Bronchial mucosa: the percentage of CD8+ T cells was increased in COPD- exS-ics and S compared to HNS. A significantly higher proportion of CD8+<br><br>T cells producing TNF-a in all COPD and S compared to HNS. The percentage of CD8+ T cells producing IFN-g and TNF-a was significantly lower in COPD-CS-ics than COPD-CS<br><br>Peripheral blood: there was a ssignificantly higher number of CD8+ T cells in COPD-CS and COPD-exS compared to HNS. The proportion of CD8+ T cells producing IFN-g and TNF-a was significantly higher in COPD-CS and COPD-exS than S and HNS
