# Supplementary material for: The role of CD8 + T lymphocytes in chronic obstructive pulmonary disease: a systematic review
Source: Inflamm Res. 2020 Oct 10;70(1):11–8. doi: 10.1007/s00011-020-01408-z (PMC7806561; doi:10.1007/s00011-020-01408-z)
Supplement: Supplementary file 3 — Supplementary file3 (PDF 86 kb) [file 11_2020_1408_MOESM3_ESM.pdf]

ONLINE RESEOURCE 3

ELECTRONIC SUPPLEMENTARY MATERIAL (ESM-3)

INFLAMMATION RESEARCH

The role of CD8+ T lymphocytes in chronic obstructive pulmonary disease: a systematic review.

Maya Williams, Ian Todd, Lucy C. Fairclough

Corresponding author: Dr Lucy C. Fairclough, School of Life Sciences, The University of Nottingham, Life Sciences Building, University Park, Nottingham NG7 2RD, United Kingdom.
Email: lucy.fairclough@nottingham.ac.uk

Table S2: Studies investigating extracellular markers and phenotype of CD8+ T lymphocytes in COPD. Eight studies were identified. Four examined expression of activation markers on CD8+ T lymphocytes in COPD, two of which also investigated killer immunoglobulin receptor expression. Cell phenotype was analysed in four studies whilst chemokine receptor expression was examined in two, as was the CD103 integrin.

Publication	Title	Subjects	COPD diagnosis	Sample	Conclusions
Roos-Engstrand et al [11]<br><br>Human 2009	Influence of smoking cessation on airway T lymphocyte Subsets in COPD	19 moderate or severe COPD, 13 smoker controls (S), 12 healthy non- smoker controls (HNS)	Global Initiative for Chronic Obstructive Lung Disease (GOLD) criteria	Bronchoalveolar lavage fluid (BAL)	The percentage of CD8+ T cells among lymphocytes was increased in COPD and S compared to HNS, and in COPD-ex smokers (exS) compared to HNS. COPD and S had increased expression of activation markers on CD8+ T cells compared to HNS, as did COPD-exS compared to HNS<br><br>Concludes that CD8+ T cells in BAL of COPD patients are highly activated regardless of current smoking status
Wang et al [12]<br><br>Human 2013	Differential Activation of Killer Cells in the Circulation and the Lung: A Study of Current Smoking status and Chronic Obstructive Pulmonary Disease (COPD)	3 cohorts: 14 COPD current smokers (CS), 10 COPD-ex smokers (exS)	American Thoracic Society guidelines	Peripheral blood	Peripheral blood: S and COPD-CS had significantly more peripheral blood CD8+ T cells which were activated ex-vivo compared to HNS. There was no significant differences in the activation of CD8+ T cells when comparing COPD-exS, S or COPD-CS. CD8+ T cell activation was positively correlated with the number of cigarettes smoked.
		9 COPD- CS, 7 COPD-exS		Peripheral blood	A lower proportion of CD8+ T cells expressed inhibitory killer immunoglobulin receptor (KIR) in COPD-CS and S compared to HNS. There was no difference in expression of activating KIRs between the groups
		5 COPD- CS, 6 COPD-exS		Induced sputum	In induced sputum, a significantly higher proportion of CD8+ T cells in COPD- CS and COPD-exS were activated ex vivo compared to HNS. A significantly higher proportion of CD8+ T cells from COPD-CS were activated compared to S.

Barceló et al [13]<br><br>Human 2008	Phenotypic characterisation of T-lymphocytes in COPD: Abnormal CD4+CD25+ regulatory T-lymphocyte response to tobacco smoking	23 moderate COPD, 29 S, 7 HNS	GOLD II-III	BAL<br>Peripheral blood	BAL: higher proportion of CD8+ CD45RA+ (naïve), and lower proportion of memory CD8+ CD45RO+ (memory) cells in COPD compared to S.<br><br>Peripheral blood: the percentage of naïve and memory CD8+ T cells was not significantly different between groups.<br><br>In BAL, the percentage of memory CD8+ T cells was greater than in peripheral blood whereas the percentage of naïve CD8+ T cells was lower than in peripheral blood<br><br>Concludes that the final maturation-activation state of CD8+ T cells in COPD is CD45RA+
Glader et al [14]<br><br>Human 2005	αeβ7 expression on CD8+ T-cells in COPD BAL fluid and on TGF-β stimulated T-cells in vitro	5 COPD, 5 HNS	GOLD	BAL<br>Peripheral blood	There was no significant difference in the expression of CD103 on CD8+ T cells from patients with COPD and HNS in either peripheral blood or BAL. Very few BAL CD8+ T cells stained positive for CD25 in either COPD or HNS whereas CD45R0 expression was high in both, so there was no significant differences between the groups. Most CD8+ T cells in BAL were CD45R0+ CD25- suggesting that the cells have previously been activated and are of the memory phenotype<br><br>In the presence of monocytes, TGF-b1 was shown to induce CD103 expression on CD8+ T cells. This may be mediated by interaction between ICAM-1 on monocytes and LFA-1 on T cells

Smyth et al [15]<br><br>Human 2008	CD8 Chemokine receptors in chronic obstructive pulmonary disease	15 COPD, 11 S, 8 HNS	COPD diagnosis made on the basis of >10 pack year, symptoms, airflow obstruction (FEV_1 < 80% predicted and FEV_1/FVC < 0.7)	BAL	COPD patients had a significantly greater number of CD8+ T cells per ml BAL compared to HNS<br>Proportion of CD8+ T cells expressing CCR3 was significantly greater in COPD and S compared with HNS and the same was seen for CCR5. There was no difference in CCR3 and CCR5 between COPD and S.<br><br>When data from COPD and S groups was combined, CCR5 expression on CD8+ T cells in BAL of current smokers was significantly higher compared to ex-smokers. There was a positive correlation between BAL CD8+ CCR5 expression and pack year history.<br><br>Lung explants – there was a significantly higher level of CCL5 in COPD compared to S. Stimulation with LPS had no effect on chemokine release. CCL11 production in COPD and S was low and similar, and also was not altered by LPS stimulation.
		10 COPD, 12 S, 6 HNS		BAL	
		6 COPD, 6 S		Bronchial explant	
Roos-Engstrand et al [16]<br><br>Human 2010	Cytotoxic T cells expressing the co-stimulatory receptor NKG2 D are increased in cigarette smoking and COPD	16 COPD- CS, 19 COPD- exS, 16 S, 21 HNS<br><br>Sub-study: 5 COPD-CS, 4 COPD-exS, 14 S, 9 HNS	GOLD	BAL<br><br>Endobronchial biopsy	Epithelial CD8+ T cells were higher in COPD compared to HNS but no different than S<br><br>Percentage of CD8+ T cells expressing activating KIR NKG2D was increased in COPD and S compared to HNS.<br>The percentage of activated CD8+ T cells (CD69+) was increased in COPD and S compared to HNS. CD69 fluorescence intensity was also increased in CD8+ T cells from COPD subjects and S compared to HNS. There were no differences in fluorescence intensity in NKG2D or HLA-DR on CD8+ T cells.

Koch et al [17]<br><br>Human 2007	Modification of surface antigens in blood CD8+ T lymphocytes in COPD: Effects of smoking	12 COPD- CS, 14 S, 13 HNS	National Institutes of Health/WHO<br><br>Smokers with COPD matched GOLD guidelines	Peripheral blood	Significant decrease in the percentage of CD8+/CD3+ T cells in smokers with COPD compared to HNS and S.<br>There was increased proportion of CD8+/CXCR3+ T cells in COPD-CS compared to S and HNS so this increase is smoking-independent.<br><br>The percentage of CD28+/CD8+ T cells as increased in COPD-CS and S compared to HNS.<br>There was decreased chemotactic activity of CD8+ T cells to monocyte chemoattractant protein 1 in COPD-CS and S compared to HNS.<br><br>The percentage of cytotoxic effector CD8+ T cells was significantly higher in COPD-CS and S compared to HNS, shows a smoking related increase in cytotoxicity.
Mikko et al [18]<br><br>Human 2013	Increased intraepithelia l (CD103+) CD8+ T cells in the airways of smokers with and without chronic obstructive pulmonary disease	27 COPD- CS, 11 COPD-exS, 40 S, 40 HNS	GOLD I and II	BAL<br>Peripheral blood	BAL: Proportion of BAL CD8+ T cells expressing CD103 was significantly higher in COPD-CS and S compared to HNS. COPD-CS also had significantly higher CD103+ CD8+ T cells than COPD-exS. Also, COPD-CS and S had significantly lower proportion of naïve CD27+ CD69- T cells in the CD8+ CD103+ population compared to HNS. COPD-exS had higher frequencies of these naïve cells than COPD-CS.<br><br>Peripheral blood: no difference in CD8+ CD103+ T cells between groups.

Urbanowicz et al [19]<br><br>Human 2010	Enhanced effector function of cytotoxic cells in the induced sputum of COPD patients	11 COPD, 10 S, 5 HNS	ATS guidelines	Induced sputum	Proportion of CD8+ T cells in induced sputum was significantly higher in COPD than S and HNS<br>COPD subjects had increased proportion of memory cells (CD45RO+RA-) and a reduction in the proportion of naïve cells (CD45RO-RA+) compared to S and HNS<br><br>Proportion of T_{EMRA} cells (CD45RO+RA+) was higher in COPD<br>Proportion of CD8+ T cells expressing both perforin and granzyme B was significantly higher in COPD compared to HNS.<br>The proportion of CD8+ T cells expressing only perforin and no granzyme B was significantly higher in S and COPD compared to HNS<br>Proportion of CD8+ T cells expressing CXCR3 was significantly higher in COPD than S. The same pattern was seen for VLA-4
Freeman et al [20]<br><br>Human 2010	Cytotoxic potential of lung CD8 T cells increases with COPD severity and with in-vitro stimulation by IL-18 or IL-15	COPD stage: 6 (I), 12 (II), 6 (III), 9 (IV) Controls; 11 S, 3 HNS<br><br>COPD stage: 2 (I), 4 (II), 6 (III), 2 (IV) Controls; 8 S<br><br>COPD stage: 7 (I), 17 (II), 11 (III), 19 (IV) Controls: 25 S, 5 HNS	GOLD	Lung tissue	The majority of lung CD8+ T cells were short term effector memory T_{EM} cells (not terminally differentiated) in the lung.<br><br>Analysis of RNA transcripts: T bet (Tc1) transcript detection was inversely correlated with lung function. GATA-3 transcripts (Tc2) were shown to be expressed by CD8+ T cells. Lung CD8+ T cells showed little or no expression of ROR-g (Tc17) transcripts<br><br>mRNA expression for perforin and granzyme B was correlated with lung function (FEV_1% predicted) whereas FasL transcript expression did not. IL-18 receptor expression on CD8+ T cells was correlated with disease severity. Stimulation of CD8+ T cells with IL-18 and IL-12 was associated with increased IFN-g and TNF-a, highlighting a TCR independent mechanism. Stimulation with IL-15 was associated with upregulation of perforin expression by CD8+ T cells

Urbanowicz et al [21]<br><br>Human 2009	Altered effector function of peripheral cytotoxic cells in COPD	11 COPD, 17 S, 18 HNS	ATS guidelines	Peripheral blood	The proportion CD8+ T cells that expressed both perforin and granzyme B was significantly lower in COPD compared to S and HNS, as was the proportion of CD8+ T cells which only expressed granzyme B (not perforin) and those which only expressed perforin (not granzyme B)<br><br>The proportion of CD45RO+RA+ (T_{EMRA} cells) was significantly lower in COPD subjects and S compared to HNS. COPD subjects had a trend of more memory cells (CD8+ CD45RO+RA-) and reduced naïve cells (CD8+CD45RO- RA+) compared to other groups, but this did not reach significance
