# Supplementary material for: The role of CD8 + T lymphocytes in chronic obstructive pulmonary disease: a systematic review
Source: Inflamm Res. 2020 Oct 10;70(1):11–8. doi: 10.1007/s00011-020-01408-z (PMC7806561; doi:10.1007/s00011-020-01408-z)
Supplement: Supplementary file 4 — Supplementary file4 (PDF 86 kb) [file 11_2020_1408_MOESM4_ESM.pdf]

ONLINE RESEOURCE 4

ELECTRONIC SUPPLEMENTARY MATERIAL (ESM-4)

INFLAMMATION RESEARCH

The role of CD8+ T lymphocytes in chronic obstructive pulmonary disease: a systematic review.

Maya Williams, Ian Todd, Lucy C. Fairclough

Corresponding author: Dr Lucy C. Fairclough, School of Life Sciences, The University of Nottingham, Life Sciences Building, University Park, Nottingham NG7 2RD, United Kingdom.
Email: lucy.fairclough@nottingham.ac.uk

Table S3: Studies investigating the inflammatory cytokine profile of CD8+ T lymphocytes in COPD. Eleven studies were identified. Nine investigated the Tc1 CD8+ T lymphocyte population, five of which also examined the Tc2 subset. Two studies highlighted the Tc1/Tc2 ratio in COPD whilst the Tc17 subpopulation was investigated in three studies.

Publication	Title	Subjects	COPD diagnosis	Sample	Conclusions
Chang et al [22]<br><br>Human 2011	CD8 positive T cells express IL-17 in patients with chronic obstructive pulmonary disease Systemic CD4+ and CD8+ T-cell cytokine profiles correlate with GOLD stage in stable COPD	16 COPD, 15 non- smokers	Global Initiative for Chronic Obstructive Lung Disease (GOLD) stage I-IV	Endobronchial biopsy	CD8+ T cells from patients with COPD expressed IL-17A and IL-17F using immunoreactivity, and were found to be the principal cells producing these cytokines.<br>RT-PCR confirmed that CD8+ T cells are capable of expressing mRNA for IL- 17A and IL-17F<br><br>There was significantly higher levels of IL-17A and IL-17F in COPD groups than controls
Paats et al [23]<br><br>Human 2012	Systemic CD4+ and CD8+ T-cell cytokine profiles correlate with GOLD stage in stable COPD	30 COPD, 10 healthy non-smokers (HNS)	GOLD II-IV	Peripheral blood	The proportion of IFN-g and TNF-a producing CD8+ T cells was significantly increased in COPD compared to HNS. patients with GOLD IV had lower proportions of IFN-g and TNF-a producing CD8+ T cells than those with GOLD II.<br><br>Proportion of IL-4 producing CD8+ T cells was low but there was a trend towards higher proportions in COPD<br>The proportion of IL-17A, IL-17F CD8+ T cells were negligible and no different between groups<br>Memory fraction of CD8+ T cells (CD45RA- CD45RO+) were main cytokine producers
Xu et al [24]<br><br>Human 2016	Peripheral Tc17 and Tc17/IFN-g cells are increased and associated with lung function in patients with COPD	25 COPD, 23 smoker controls (S), 15 HNS	GOLD II-IV	Peripheral blood	The proportion of Tc1 cells amongst CD8+ T cells was significantly higher in COPD compared to S and HNS.<br>The percentage of Tc17 cells amongst CD8+ T cells was increased in COPD compared to S and HNS. Among Tc17 cells, the percentage of Tc17/IFN-g cells was higher in COPD than S and HNS. The higher frequency of Tc17/IFN-g cells in COPD was inversely correlated with lung function

Zhu et al [25]<br><br>Human<br><br>2009	Peripheral T cell Functions Correlated with the Severity of Chronic Obstructive Pulmonary Disease	81 COPD, 22 HNS	GOLD	Peripheral blood	Production of IFN-g by CD8+ T cells was more frequent in COPD than normal, and was directly associated with disease severity. IL-4 production was more frequent in CD8+ T cells from COPD than HNS CD8+ T cells from COPD patients were more frequently activated than those from HNS (measured by HLA-DR expression)<br><br>Continued smoking was associated with tendencies to blunt cytokine elaborations and CD8+ T cell activation
Shirai et al [26]<br><br>Human 2010	Correlation between peripheral blood T-cell profiles and clinical inflammatory parameters in stable COPD	31 COPD, 14 S, 16 ex-smoker controls (exS)	GOLD	Peripheral blood	The proportion of IFN-g producing CD8+ T cells was significantly higher in COPD than controls but there was no difference in production within the COPD group when compared by disease severity or current smoking status<br>The proportion of IL-4 producing CD8+ T cells in COPD was significantly higher than controls but again, no difference within the COPD group when compared by disease severity or smoking status<br>No difference in the ratio of IFN-g/IL-4 producing CD8+ T cells between the COPD and control groups
Nadigel et al [27]<br><br>Human 2011	Cigarette smoke increases TLR4 and TLR9 expression and induces cytokine production from CD8+ T cells in chronic obstructive pulmonary disease	8 COPD, 5 non/ex- smoker controls<br><br>9 COPD, 8 non/ex- smoker controls	GOLD	Endobronchial biopsy<br><br>Peripheral blood	Lung: there was a significant increase in the percentage of CD8+ T cells expressing TLR4 and TLR9 in COPD (90%) compared to controls (20%)<br><br>Peripheral blood: the percentage of CD8+ T cells expressing TLR4 and TLR9 was no different between groups and was relatively low<br>Peripheral blood CD8+ T cells were treated with cigarette smoke condensate (CSC) which was associated with increased TLR protein but not mRNA expression CSC also induced cytokine production from CD8+ T cells (IL-6, IL-1b, IL-10, IL-12p70, IFN-g and TNF-a), potentially through activation of TLRs<br>TLR inhibitors were used to treat CD8+ T cells. TLR9 inhibition lead to a decrease in CSC-induced TNF-a production

Yu et al [28]<br><br>Human 2013	CD8+ Tc-lymphocyte immunodeviation in peripheral blood and airway from patients of chronic obstructive pulmonary disease and changes after short-term smoking cessation	14 COPD current smokers (CS), 16 S, 12 HNS	GOLD I-II	BAL Peripheral blood	Significantly higher Tc1/Tc2 cell ratio in COPD patients than S and HNS, and also between S and HNS<br>In COPD, the proportion of CD8+ T cells and Tc1/Tc2 ratio was negatively correlated with lung function (FEV_1%predicted). Tc1/Tc2 also had a negative correlation with FEV_1/FVC<br><br>Short term smoking cessation did not significantly affect the Tc1/Tc2 ratio, although it was slightly lower, so in COPD in general there is a bias towards Tc1 – shown in peripheral blood and induced sputum.
		10 quitting, 9 continuing smokers with COPD		Induced sputum Peripheral blood	
Barceló et al [29]<br><br>Human 2006	Intracellular cytokine profile of T lymphocytes in patients with chronic obstructive pulmonary disease	14 COPD, 16 S, 7 HNS	Not specified	BAL Peripheral blood	BAL: CD8+ T cells from COPD produced more cytokines than those in BAL from controls<br>The CD8+ T cells from COPD showed mainly a Tc2 cytokine profile since they had significantly higher production of IL-2, IL-4, IL-10 and IL-13 than S. Positive staining for Tc2 cells was negatively correlated with FEV_1<br><br>Tc1 cytokines were not correlated with lung function<br><br>Peripheral blood: there was no difference in CD8+ T cell intracellular cytokine expression between groups
Barczyk et al [30]<br><br>Human 2006	Cytokine production by bronchoalveolar lavage T lymphocytes in chronic obstructive pulmonary disease	11 COPD, 9 controls	FEV_1/FVC < 70% (GOLD not specified)	BAL	BAL: higher percentage of CD8+ T cells expressing IFN-g and TNF-a than just IFN-g alone in all subjects. The percentage of BAL CD8+ T cells expressing IL- 4 was significantly higher in COPD than controls. A higher percentage of CD8+ T cells produced IL-4 than CD4+ T cells suggesting a greater Tc2 than Th2 response whereas IFN-g was produced by more CD4+ T cells than CD8+. CD8+ T cells were cultured and stimulated which increased the expression of IFN-g and TNF-a and also IL-4. Peripheral blood: the number of CD8+ T cells expressing IL-4 was less than in BAL, whereas the percentage of Tc1 cells was greater in peripheral blood than BAL. The cells were not affected by stimulation
		4 COPD, 4 S		Peripheral blood	

Eppert et al [31]<br><br>Mouse 2013	Functional Characterisation of T cell Populations in a Mouse Model of Chronic Obstructive Pulmonary Disease	Female Balb/cJ and C57B6 WT mice C57B6 Ciita$^{-/-}$ and C57B6 B2m$^{-/-}$ exposed to filtered air (FA) or cigarette smoke for 4h/day, 5d/wk, 24 weeks	N/A	BAL<br>Lung tissue	The proportion of CD8+ T cells that stained positive for IFN-g and TNF-a was higher from the lungs of smoke exposed mice than FA exposed. CD8+ T cells from the lungs of smoke exposed mice produced more IFN-g than those from FA mice, shows that cigarette smoke induces pro-inflammatory phenotype. CD8+ T cells from smoke exposed donors were sufficient to cause pulmonary inflammation, assessed by total cell number retrieved from BAL but not histological examination. They were not capable of causing alveolar destruction or increased leucocytic accumulation.<br><br>Transfer of CD8+ T cells from donor mice to naïve recipient mice which were deficient in either MHC class I or II showed that antigen presentation to CD8+ T cells on MHC class I molecules contributes to the transfer of airspace enlargement and inflammation
Lethbridge et al [32]<br><br>Human 2010	A novel technique to explore the functions of bronchial mucosal T cells in chronic obstructive pulmonary disease: application to cytotoxicity and cytokine immunoreactivity	15 COPD- CS, 12 COPD-exS, 11 S, 10 HNS	GOLD	Endobronchial biopsy	Significantly greater percentage of CD8+ T cells from COPD-CS expressed IFN-g and TNF-a and IL-13 immunoreactivity compared to controls<br>The median percentage of CD8+ T cells expressing IFN-g and TNF-a was lower in COPD-exS and both control groups. The percentage of CD8+ T cells expressing IL-13 was elevated in COPD-exS compared to controls.
