# Supplementary material for: The role of CD8 + T lymphocytes in chronic obstructive pulmonary disease: a systematic review
Source: Inflamm Res. 2020 Oct 10;70(1):11–8. doi: 10.1007/s00011-020-01408-z (PMC7806561; doi:10.1007/s00011-020-01408-z)
Supplement: Supplementary file 5 — Supplementary file5 (PDF 72 kb) [file 11_2020_1408_MOESM5_ESM.pdf]

ONLINE RESEOURCE 5

ELECTRONIC SUPPLEMENTARY MATERIAL (ESM-5)

INFLAMMATION RESEARCH

The role of CD8+ T lymphocytes in chronic obstructive pulmonary disease: a systematic review.

Maya Williams, Ian Todd, Lucy C. Fairclough

Corresponding author: Dr Lucy C. Fairclough, School of Life Sciences, The University of Nottingham, Life Sciences Building, University Park, Nottingham NG7 2RD, United Kingdom.
Email: lucy.fairclough@nottingham.ac.uk

Table S4: Studies investigating the cytotoxic function of CD8+ T lymphocytes in COPD. Ten studies were identified. Seven investigated the expression of cytotoxic proteins by CD8+ T lymphocytes in COPD whilst the Fas/FasL pathway was examined in three. Expression of the programmed cell death protein 1 (PD-1) on CD8+ T lymphocytes in COPD was highlighted in one study.

Publication	Title	Subjects	COPD diagnosis	Sample	Conclusions
Kim et al [33]<br><br>Human 2013	A possible role for CD8+ and non-CD8+ cell granzyme B in early small airway remodeling in centrilobular emphysema	14 panlobular emphysema (PLE), 32 centrilobular emphysema (CLE), 13 HNS	Emphysema diagnosis was made based on histological exam not all patients with PLE or CLE qualified for COPD Global Initiative for Chronic Obstructive Lung Disease (GOLD) criteria	Lung tissue	Volume fraction (Vv) of CD8+ T cells in small airway wall was greater in CLE than control and PLE, as was the Vv of granzyme B positive cells<br>Vv of granzyme B positive cells was greater than that of CD8+ T cells in CLE subjects, and not all CD8+ T cells were positive for granzyme B on dual staining.<br><br>There were other granzyme B positive cells – monocytes and granulocytes
Vernooy et al [34]<br><br>Human 2007	Increased Granzyme A expression in Type II Pneumocytes of Patients with Severe Chronic Obstructive Pulmonary Disease	22 COPD, 15 controls	GOLD IV	Lung tissue	Granular staining for granzyme A was noted in cytotoxic lymphocytes and the same was true for granzyme B<br>However, granzyme A and granzyme B staining was also observed in type II pneumocytes and alveolar macrophages (resident lung cells)<br><br>CD8+ T cells are activated in peripheral lung tissue since they express serine proteases
Shiratsuchi [35]<br><br>Human 2011	Measurement of soluble perforin, a marker of CD8+ T lymphocyte activation in epithelial lining fluid	30 COPD, 11 ex- smokers, 10 non-smokers	GOLD	Epithelial lining fluid (central and peripheral airways)	Levels of perforin were significantly higher in COPD than in non-smokers and ex-smokers for both central and peripheral airways<br>There was no significant correlation between smoking status and perforin levels in either central or peripheral airways in COPD patients<br><br>Perforin level in peripheral airways was closely correlated with lung function (FEV_1, FEV_1/FVC and lung diffusion capacity)

Domagala-Kulawik et al [36]<br><br>Human<br><br>2007	Increased proportion of Fas positive CD8+ cells in peripheral blood of patients with COPD	18 COPD, 12 smoker controls (S), 12 healthy non- smokers (HNS)	American Thoracic Society (ATS)/ European Respiratory Society guidelines	Peripheral blood	Elevated proportion of CD8+ cells expressing Fas (CD95) in COPD groups compared to controls. The proportion of CD8+ T cells positive for CD95 as a percentage of all lymphocytes was increased in COPD compared to controls. The proportion of Fas positive CD8+ T cells was significantly correlated with reduced lung function (FEV_1/FVC) and hypoxaemia (arterial blood oxygen pressure) The proportion of Fas positive CD8+ T cells was significantly correlated with mean pack years smoked in the whole smoking group, but not with only COPD
Domagala-Kulawik et al [37]<br><br>Human<br>2011	Fas+ lymphocytes and CD4+/CD25+ cells in peripheral blood of never smoking patients with chronic obstructive pulmonary disease	12 never smokers with COPD, 18 smokers with COPD, 12 S, 12 HNS	Recommendations of the Polish society of lung diseases about diagnosis and therapy of COPD	Peripheral blood	The proportion of Fas positive CD8+ T cells in both never-smokers and smokers with COPD was significantly higher than healthy controls. This shows a disease related increase in Fas expression. There was no difference in the proportion of CD8+/Fas+ lymphocytes between the never-smoking and current smoking COPD groups.
McKendry et al [38]<br><br>Human<br>2016	Dysregulation of antiviral function of CD8+ T cells in the chronic obstructive pulmonary disease lung. Role of the PD-1 – PD-L1 Axis	33 COPD (1 never, 20 ex-, 12 current smokers), 24 controls (6 never, 15 ex-, 3 current smokers)	Not specified	Lung tissue	A significantly higher proportion of CD8+ T cells from COPD expressed programmed cell death protein 1 (PD-1) than cells from controls.<br>Ex-vivo model of influenza X31 infection. PD-1 expression was upregulated on CD8+ T cells in both control and COPD explants in response to X31 infection.<br><br>The percentage of CD8+ T cells from control subjects and patients with COPD expressing PD-1 was increased in response to X31.<br>CD8+ T cells from controls significantly upregulated CD107a in response to viral infection whereas this did not happen in CD8+ T cells derived from COPD explants. CD107a is a degranulation marker, so these results conclude that CD8+ T cells in the lung may have an impaired degranulation response to viral infection

Hodge et al [39]<br><br>Human and Mouse<br><br>2011	Role of increased CD8/CD28 null T cells and alternative co-stimulatory molecules in chronic obstructive pulmonary disease	30 COPD- CS, 18 COPD-exS, 34 S, 15 HNS<br><br>Mice – 10 smoke exposed, 6 sham exposed for 12 weeks	GOLD	Peripheral blood<br><br>Mice – Bronchoalveolar lavage (BAL), lung tissue, blood	There were significantly increased CD8/CD28^{null} T cells in the peripheral blood of COPD-CS and COPD-exS compared to controls Increased expression of 4-1BB in both COPD groups on CD8+ T cells. CTLA4 was increased on CD8+ T cells from S and COPD-CS. OX40 was significantly increased on CD8+ T cells from S and COPD-CS. There were no significant differences in CD40L<br><br>CD8/CD28^{null} cells expressed significantly more IFN-g, granzyme and perforin than CD8/CD28+ T cells. They also expressed significantly more OX40, 4-1BB, CTLA4 when stimulated than CD8/CD28+ T cells<br>Mice: no significant differences in the percentage of CD8+ T cells in any compartment following 12 weeks of smoke exposure. However there was a significant increase in CD8/CD28^{null} T cells in airway (BAL) and trend for an increase in lung and peripheral blood.
