# Supplementary material for: The role of CD8 + T lymphocytes in chronic obstructive pulmonary disease: a systematic review
Source: Inflamm Res. 2020 Oct 10;70(1):11–8. doi: 10.1007/s00011-020-01408-z (PMC7806561; doi:10.1007/s00011-020-01408-z)
Supplement: Supplementary file 6 — Supplementary file6 (PDF 77 kb) [file 11_2020_1408_MOESM6_ESM.pdf]

ONLINE RESEOURCE 6

ELECTRONIC SUPPLEMENTARY MATERIAL (ESM-6)

INFLAMMATION RESEARCH

The role of CD8+ T lymphocytes in chronic obstructive pulmonary disease: a systematic review.

Maya Williams, Ian Todd, Lucy C. Fairclough

Corresponding author: Dr Lucy C. Fairclough, School of Life Sciences, The University of Nottingham, Life Sciences Building, University Park, Nottingham NG7 2RD, United Kingdom.
Email: lucy.fairclough@nottingham.ac.uk

Table S5: Studies investigating effector function of CD8+ T lymphocytes in COPD. Five studies were identified, four of which were human and one mouse. The effects of CD8+ T lymphocytes on mediating pulmonary inflammation was investigated in three studies whereas dysfunction of the T cell receptor was highlighted in two studies.

Publication	Title	Subjects	COPD diagnoss	Sample	Conclusions
Motz et al [40]<br><br>Mouse 2008	Persistence of lung CD8 T Cell Oligoclonal Expansions upon Smoking Cessation in a Mouse Model of Cigarette Smoke-Induced Emphysema	Mice:<br>BALB/cJ mice. Mice were acclimated to smoke exposure for 1 week before study initiation Mice were exposed for 4 hours a day, 5 days a week for 6 months<br>An additional cohort were exposed for 6 months and then housed unexposed for an additional 6 months Age-matched filtered air exposed mice were used as controls	N/A	Lung tissue	Concludes that oligoclonal T cell expansions occur primarily in the CD8 TCR repertoire. Antigenic activation of CD8+ T cells could play a role in COPD pathogenesis.<br>Oligoclonal expansions occurred in the CD8 TCR Vb repertoire of cigarette- smoke exposed mice. A significant number of oligoclonal expansions occurred at common CDR3 lengths among cigarette smoke exposed mice which suggests a common antigenic stimulus<br><br>Analyses on the spleen CD8+ T cells from the same mice showed no skewing in the CDR3 distributions (which shows specific TCR usage). Therefore, the specific micro-environment of the lung generates unique antigens driving oligoclonal expansions as a consequence of chronic cigarette smoke exposure The lung TCR repertoire was also oligoclonal in mice which underwent smoke cessation, showing that oligoclonal expansions persist despite cessation of smoking
Podolin et al [41]<br><br>Mouse 2013	T cell depletion protects against alveolar destruction due to chronic cigarette smoke exposure in mice	Mice: female C57BL/6 mice were exposed to 4% cigarette smoke for 2h/day, 5d/week for up to 24 weeks. 8 were in the treatment group For prophylactic studies, antibodies were given from 2 weeks before the smoke exposure and continued throughout the 24 weeks<br><br>For therapeutic studies, antibodies were given from 12 weeks to 24 weeks of smoke exposure	N/A	BAL<br>Lung tissue	When 3 T-cell depleting antibodies were used, there was 76% protection against alveolar destruction. When using the CD8 depleting antibody alone, there was still a significant protection (56%) against alveolar destruction as measured by mean linear intercept. This suggests that CD8+ T cells may require CD4+ T cells to have full effects.<br><br>Specific depletion of CD8+ T cells offered a similar level of protection from alveolar destruction as treatment with cyclosporin A.<br>Protection was associated with reduced numbers of infiltrating neutrophils, so the CD8+ T cells may facilitate neutrophil recruitment to the bronchoalveolar space.

Borchers et al [42]<br><br>Mouse 2007	CD8+ T cells contribute to macrophage accumulation and airspace enlargement following repeated irritant exposure	Mice: wild type C57BL/6J mice, and mice deficient in CD8+ T cells (*Cd8*$^{(-/-)}$) were exposed to filtered air or 2.0ppm acrolein 6h/d, 5d/w for up to 12 weeks	N/A	BAL<br>Lung tissue	Following repeated toxicant exposure, CD8+ T cell number increases in the lungs of wild type mice. CD8+ T cells are primarily localized to the parenchyma, although some were in the submucosa of the conducting airways<br>Wild type mice exposed to acrolein saw an increase in macrophage accumulation in BAL. This was not noted In (*Cd8*$^{(-/-)}$) mice exposed to acrolein Progressive destruction of the alveolar walls due to acrolein was attenuated in (*Cd8*$^{(-/-)}$) mice exposed to acrolein. The increased mean linear intercept was greater in wild-type mice compared to the (*Cd8*$^{(-/-)}$) mice exposed to acrolein after 12 weeks.<br>CD8+ T cells play a role in acrolein-induced IP-10 and IFN-g expression RANTES and MCP-1 were increased in wild type and *(Cd8*$^{(-/-)}$) mice, but the increase was significantly greater in wild type than in *(Cd8*$^{(-/-)}$) mice MMP2 and MMP9 were significantly increased in the lung of wild type, but not<br>in *(Cd8*$^{(-/-)}$) mice
Maeno et al [43]<br><br>Mouse 2007	CD8+ T cells are required for<br>Inflammati-on and destruction in cigarette-smoked induced emphysema in mice	Mice: wild-type C57BL/6J mice, CD8+ T cell deficient (CD8$^{-/-}$) mice and CD4+ T cell deficient (CD4$^{- /-}$) mice<br><br>Groups were subjected to the smoker of 2 unfiltered cigarettes per day, 6 days a week for 6 months	N/A	BAL<br>Lung tissue	There were increased CD8+ T cells in the lungs of WT smoke exposed mice compared to non-smoke exposed controls, suggesting that smoke induces an increase in CD8+ T cells<br>In the absence of CD8+ T cells, there is reduced inflammation in response to long term cigarette smoke exposure in mice.<br><br>In WT mice there was a significant accumulation of macrophages and neutrophils in BAL after cigarette smoke exposure, but there was no change in any subset of immune cells in CD8$^{-/-}$ mice.<br>Mean linear intercept was not changed in CD8$^{-/-}$ mice in response to cigarette smoke, whereas there was a significant increase in WT mice in response to cigarette smoke<br>In smoke-exposed CD8$^{-/-}$ mice there was no increased IP-10 production and minimal expression of MMP-12.<br>In WT mice, exposure to cigarette smoke lead to increased chemotactic elastin fragments, but this did not happen in CD8$^{-/-}$ mice<br>Lungs of CD8$^{-/-}$ mice exposed to cigarette smoke have reduced monocyte chemotactic activity

Grundy et al [44]<br><br>Human 2013	Down regulation of T Cell Receptor expression in COPD pulmonary CD8 cells	6 COPD, 6 smokers (S)<br><br>7 COPD, 5 S, 8 healthy non-smokers (HNS)	Not specified	Lung tissue Periph-eral blood<br><br>Broncho-alveolar lavage (BAL)	Expression of genes related to the T cell receptor (TCR) signaling pathway were downregulated in the pulmonary samples when compared to peripheral blood samples. Expression of these genes was lower by COPD pulmonary CD8+ T cells than S pulmonary CD8+ T cells but this was not significant.<br><br>A lower proportion of CD8+ T cells from COPD were positively stained for CD247 compared to S and HNS. Downregulation of CD247, a gene involved in signaling and anchoring of the TCR, has been associated with autoimmune disease.<br>Concludes that there is downregulation of the TCR associated effector function in COPD CD8+ T cells. The TCR is responsible for antigen specific responses, so there may be non-antigen specific activation of CD8+ T cells which explains their increased effector function
