# Supplementary material for: The role of CD8 + T lymphocytes in chronic obstructive pulmonary disease: a systematic review
Source: Inflamm Res. 2020 Oct 10;70(1):11–8. doi: 10.1007/s00011-020-01408-z (PMC7806561; doi:10.1007/s00011-020-01408-z)
Supplement: Supplementary file 7 — Supplementary file7 (PDF 67 kb) [file 11_2020_1408_MOESM7_ESM.pdf]

**ONLINE RESEOURCE 7**

**ELECTRONIC SUPPLEMENTARY MATERIAL (ESM-7)**

**INFLAMMATION RESEARCH**

# The role of CD8+ T lymphocytes in chronic obstructive pulmonary disease: a systematic review.

**Maya Williams, Ian Todd, Lucy C. Fairclough**

**Corresponding author: Dr Lucy C. Fairclough, School of Life Sciences, The University of Nottingham, Life Sciences Building, University Park, Nottingham NG7 2RD, United Kingdom.**
**Email: lucy.fairclough@nottingham.ac.uk**

**Table S6: Studies investigating the role of CD8+ T lymphocytes in acute exacerbations of COPD.** Four studies investigated CD8+ T lymphocytes at acute exacerbation of COPD, three of which examined the CD8+ T lymphocyte subpopulations.

| Publication | Title | Subjects | COPD diagnosis | Sample | Conclusions |
|---|---|---|---|---|---|
| Chen et al [45]<br><br>Human 2016 | Imbalance between subsets of CD8+ peripheral blood T cells in patients with chronic obstructive pulmonary disease | 24 stable COPD (SCOPD), 14 acute exacerbation of COPD (AECOPD), 14 healthy non-smokers (HNS) | Global Initiative for Chronic Obstructive Lung Disease (GOLD) criteria<br><br>AECOPD – symptoms diagnostic for COPD exacerbation without any new therapeutic intervention | Peripheral blood | A higher percentage of CD8+ T cells in peripheral blood of AECOPD patients than the other two groups. The proportions in SCOPD and HNS were similar.<br>Tc1 cells were elevated in AECOPD and SCOPD compared to HNS and were the predominant CD8+ T cell subset. Tc17 cells were also increased at AECOPD.<br>Tc2 cells were reduced in SCOPD compared to AECOPD and controls<br>The anti-inflammatory CD8+ T cells (Tc10) were decreased in both COPD groups compared to controls. |
| Freeman et al [46]<br><br>Human 2015 | Acute exacerbations of chronic obstructive pulmonary disease associated with decreased CD4+ and CD8+ T cells and increased growth & differentiation factor-15 (GDF-15) in peripheral blood | Subjects with COPD and history of previous AE- COPD were followed up for up to 3 years.<br><br>Of 33 enrolled subjects, 13 participated in multiple stable visits and had at least 1 AE- COPD visit, which generated 18 total AE-COPD events | GOLD<br><br>AECOPD – change above baseline in respiratory symptoms plus addition by a study physician of antibiotics, oral steroids, review of history, physical examination, chest radiographs<br><br>AECOPD diagnosis was made prior to collection of samples | Peripheral blood<br>Sputum | In AECOPD there is a marked decrease in the absolute number and the percentage of CD8+ T cells in the peripheral blood compared to the relative stable visit. This is followed by a rebound back to normal levels at the next stable visit<br><br>CD8+ T cells correlated significantly and inversely with CRP (inflammatory marker)<br>In some patients whose stable visit was shortly before their AECOPD, the decline in cell frequency could be observed before the exacerbation – therefore, immune activation may precede symptoms |

| | | | | | |
|---|---|---|---|---|---|
| Makris et al [47]<br><br>Human 2008 | Tc2 response at the onset of COPD exacerbations | 24 COPD examined at the onset of exacerbation and in stable condition | GOLD<br><br>Initiation of symptoms diagnostic for COPD exacerbation in past<br><br>72h, abstention from any new therapeutic intervention, absence of signs suggestive of severe exacerbation requiring hospitalisation | Induced sputum taken at exacerbation and at stable condition (>8 weeks after exacerbation) | Percentage of CD8+ T cells amongst lymphocytes was significantly increased during exacerbations compared to stable condition<br>The percentage of CD8+ IFN-g+ cells was no different at stable or exacerbation whereas the percentage of CD8+ IL-4+ cells was significantly increased, suggesting that the cells are polarized towards Tc2 profile at AECOPD<br>No statistically significant relationship between CD8+ T cell subpopulation ratios and exacerbation type, age, sex, pack years, smoking status or $FEV_1$% predicted change between exacerbation and stable condition |
| Tsoumakidou et al [48]<br><br>Human 2005 | Changes in sputum T-lymphocyte subpopulation s at the onset of severe exacerbations of chronic obstructive pulmonary disease | 12 COPD at onset of acute severe exacerbation and on stable condition | GOLD<br><br>Severe exacerbation – use of accessory respiratory muscles, paradoxical chest wall movement, respiratory failure breathing room air and/or requiring hospital admission | Induced sputum taken at exacerbation and 16 weeks after remission | There was a lower CD4/CD8 + T cell ratio at the onset of exacerbation which suggests that there are more CD8+ T cells. There is also a lower CD8+ IFN-g/CD8+ IL-4 positive cell ratio at the onset of exacerbation which suggests that there is a Tc2 mediated immune response at the onset of severe COPD exacerbation. |
